# Impact of Pulmonary Hypertension and Patent Ductus Arteriosus in Preterm Infants with Presumed Pulmonary Hypoplasia †

**DOI:** 10.3390/biomedicines13071725

**Published:** 2025-07-15

**Authors:** Sol Kim, Yumi Seo, Moon-Yeon Oh, Min Soo Kim, Sook Kyung Yum

**Affiliations:** 1 Department of Pediatrics, Seoul St. Mary’s Hospital, College of Medicine, The Catholic University of Korea, Seoul 06591, Republic of Korea; 2 Department of Pediatrics, Incheon St. Mary’s Hospital, College of Medicine, The Catholic University of Korea, Seoul 06591, Republic of Korea

**Keywords:** patent ductus arteriosus, preterm premature rupture of membrane, pulmonary circulation, pulmonary hypertension, very low birth weight

## Abstract

**Objectives**: Pulmonary hypertension and hemodynamically significant PDA (hsPDA) involve seemingly opposite physiological features—decreased pulmonary blood flow and pulmonary overcirculation, respectively—but the literature demonstrates variable respiratory consequences in association with each of these morbidities. The aim of this study is to evaluate whether the two factors representing pulmonary circulation provide different contributions to respiratory outcomes in preterm infants with and without pulmonary hypoplasia. **Methods**: The medical records of preterm very low birth weight (VLBW) infants admitted to our unit during the study period from January 2013 to December 2020 were retrospectively reviewed. Preterm VLBW infants were divided into groups according to the presence of presumed pulmonary hypoplasia (PPH). Multivariable logistic regression analysis was performed to assess the association of PPH and pulmonary hypertension or delayed PDA closure with in-hospital outcomes. **Results**: Postnatal age at final treatment for PDA was significantly later [median 33 vs. 19 days, *p* = 0.025] in the PPH group. Multivariable analysis indicated that early pulmonary hypertension was significantly associated with neonatal death [aOR (95%CI) 11.575 (2.988–44.833) for no-PPH vs. 9.981 (1.334–74.647) for PPH]. Delayed PDA closure was associated with increased odds of adverse respiratory and composite outcomes [aOR (95%CI) 4.929 (1.613–15.055) and 3.320 (1.048–10.515), respectively] but decreased odds of neonatal death in the no-PPH group. However, Cox proportional hazards models did not demonstrate statistically significant associations for PPH, early pulmonary hypertension, or delayed PDA closure with mortality, likely due to time-varying effects and the absence of death events in the subgroup of infants with both PPH and delayed PDA closure. **Conclusions**: PPH is associated with a higher prevalence of air leak syndrome and pulmonary hypertension. Delayed PDA closure exerts different effects on respiratory outcomes in preterm VLBW infants with and without PPH. Although early pulmonary hypertension appears to be a key circulatory factor contributing to neonatal death, its effect may vary over time. These findings underscore the importance of accounting for time-dependent effects when interpreting pulmonary circulatory risk factors in clinical practice.

## 1. Introduction

Mid-trimester preterm premature rupture of membranes (PPROM) and oligohydramnios are risk factors for pulmonary underdevelopment, which is induced by altered angiogenesis and disrupted alveolarization [1,2]. Such alterations of lung volume and abnormal pulmonary vasculature in premature birth may further complicate the infant’s pulmonary circulatory status, leading to increased pulmonary vascular resistance [3]. In affected infants, the clinical manifestations of pulmonary hypertension, such as labile or consistent hypoxemia, can be alleviated using pulmonary vasodilators such as inhaled nitric oxide [4]. Echocardiographic findings of pulmonary hypertension include tricuspid regurgitation and/or interventricular septum flattening, bidirectional or right-to-left shunt at the atrial level, or via patent ductus arteriosus (PDA) [4].

Conversely, a left-to-right shunt through the PDA, commonly observed in preterm infants, increases pulmonary blood flow. When the shunt is large, it can lead to pulmonary overcirculation and congestion, as well as decreased systemic perfusion. Clinical manifestations include feeding intolerance, hypotension, and oliguria [5]. Such cases are referred to as “hemodynamically significant PDA (hsPDA)” and carry a risk of congestive heart failure.

Furthermore, sustained pulmonary overcirculation imposes shear stress on the pulmonary endothelium, leading to vascular injury and remodeling. These structural changes, including intimal proliferation and medial hypertrophy, result in increased pulmonary vascular resistance. Chronic exposure to hsPDA may thus contribute to a gradual rise in pulmonary vascular resistance, eventually leading to the development of pulmonary hypertension. As pulmonary hypertension progresses, the right heart is placed under increasing strain. In severe or prolonged cases, this may result in shunt reversal and ultimately culminate in Eisenmenger physiology [6].

Pulmonary hypertension and hsPDA involve seemingly opposite physiological features—decreased pulmonary blood flow and pulmonary overcirculation, respectively—but the literature demonstrates variable respiratory consequences in association with each of these morbidities [7,8,9,10]. Here, we sought to evaluate whether these two factors representing pulmonary circulation status contribute differently to respiratory outcomes in preterm infants depending on fetal pulmonary underdevelopment.

## 2. Materials and Methods

### 2.1. Patient Inclusion and Data Collection

The electronic medical records of preterm very low birth weight (VLBW) infants admitted to our unit (a level III NICU) during the study period from January 2013 to December 2020 were retrospectively reviewed. The included infants were divided according to the presence of presumed pulmonary hypoplasia (PPH), which was defined as (a) PPROM > 7 days and/or (b) oligohydramnios. This definition was based on the threshold time interval associated with adverse outcomes, as described in the previous literature [11,12].

**Figure 1:**
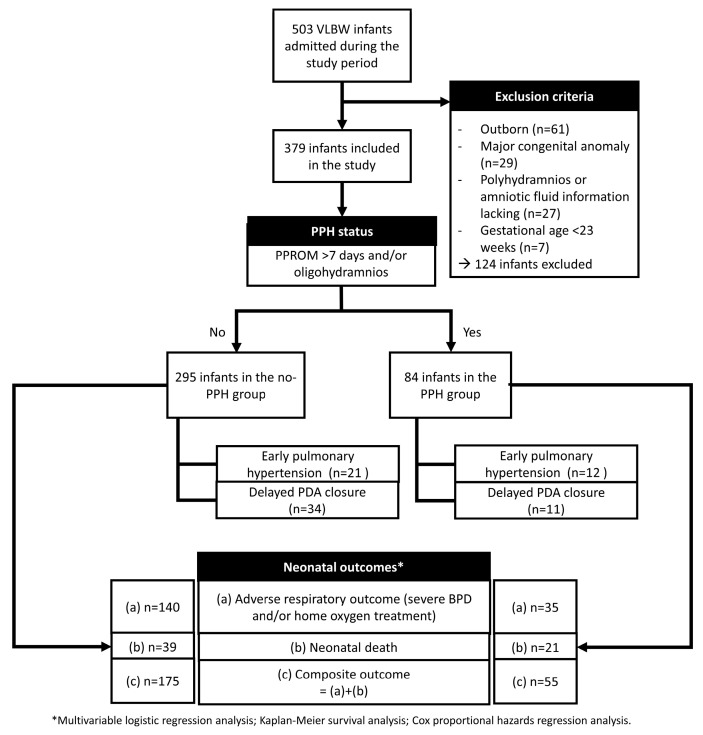
Flowchart of patient selection; group allocation based on PPH status. Descriptive outcome data (deaths and adverse outcomes) are summarized in each group for reference. Full outcome analyses are presented in the Results Section.

During the study period, 503 VLBW infants were admitted to the NICU. Of these, outborn infants (n = 61) and those with major congenital anomalies (n = 29), polyhydramnios, a lack of information concerning amniotic fluid (n = 27), and preterm infants born at less than 23 weeks of gestation (n = 7) were excluded. Figure 1 summarizes the inclusion and exclusion process, group allocation according to PPH status. A total of 379 infants (295 in the no-PPH group and 84 in the PPH group) were included. The flow also outlines descriptive outcome frequencies for each group and the statistical approaches used in the analysis.

The following information related to baseline neonatal and maternal characteristics was obtained: gestational age (GA, weeks), birth weight (BW, g), whether the infant was small for gestational age (SGA) (BW < 10th percentile based on Fenton’s growth chart), 1 and 5 min Apgar scores, sex, single/multiple births, delivery mode, maternal age (years), maternal morbidities (diabetes and hypertension), histologic chorioamnionitis, method of conception (natural or via assisted reproduction therapy), and antenatal corticosteroid administration. The onset and length of PPROM (h) and the presence of oligohydramnios were also obtained.

Data on respiratory morbidities and hsPDA treatment were collected. Respiratory distress syndrome was diagnosed when surfactant was administered via an endotracheal tube in case of failure to achieve a target SpO2 of ≥95% despite the provision of FiO2 ≥ 40%, accompanied by typical ground-glass appearance and/or air-bronchograms on chest radiographs. The number of surfactant doses administered was recorded. Air leak syndrome was defined only when interventions, such as chest tube insertion or angio-needle aspiration, were performed to relieve hypoxia and/or hypotension due to pneumothorax/pneumomediastinum. Massive pulmonary hemorrhage was diagnosed when blood from the airway was identified with concomitant or sequential cardiopulmonary collapse.

Infants with pulmonary hypertension were defined as those who presented with labile hypoxemia with or without differential hypoxia (>5–10% difference between preductal and postductal SpO2) that could not be improved by conventional treatment with appropriate ventilatory management and surfactant administration. The requirement for pulmonary vasodilator therapy (e.g., inhaled nitric oxide, milrinone infusion, sildenafil and/or bosentan administration, and iloprost nebulizer) was a requisite for the clinical diagnosis of pulmonary hypertension. Echocardiography was performed at or immediately after the diagnosis of clinical pulmonary hypertension (i.e., as soon as possible) by the pediatric cardiologist on duty. Echocardiographic diagnosis of pulmonary hypertension was based on the findings of bidirectional or right-to-left shunt at the atrial level or through PDA, tricuspid regurgitation, interventricular septum flattening, and right atrial enlargement/right ventricular hypertrophy with or without right heart dysfunction [13,14]. Early pulmonary hypertension was defined as the occurrence of pulmonary hypertension < 7 days after birth. The development of pulmonary hypertension beyond this period was defined as late pulmonary hypertension.

PDA was diagnosed via echocardiography. Hemodynamic significance was defined based on clinical symptoms and signs such as murmur, bounding pulse, blood pressure fluctuation or hypotension, increased pulse pressure, decreased urine output not attributable to underlying anatomical or functional genitourinary tract disease or etiologies other than shunt steal, exacerbating respiratory condition not attributable to pulmonary disease, and pulmonary edema or cardiomegaly on chest radiographs. Echocardiographic severity was determined based on PDA size, shunt flow pattern (pulsatile, growing, or continuous), peak systolic velocity of the shunt flow, end-diastolic velocity of the left pulmonary artery (>20 cm/s), left atrium-to-aorta ratio (>1.4), and hyperdynamic heart motion. When hsPDA was diagnosed, either medical treatment using a cyclooxygenase inhibitor (COX-I) or surgical treatment was selected. Ibuprofen (intravenous or oral) was the only drug available for the treatment of PDA in our unit during the study period. Surgical PDA ligation was performed in infants who experienced medical treatment failure and those in whom ibuprofen use was contraindicated. The surgery was performed at the bedside of the neonatal intensive care unit (NICU) by a dedicated pediatric cardiac surgical team. Data on postnatal age and corrected age at medical or surgical treatment for hsPDA were collected. The time point of PDA closure was defined based on echocardiographic confirmation of closure after medical or surgical treatment. PDA identified at ≥21 days of life was defined as “delayed PDA closure.”

Data related to the following in-hospital outcome variables were also collected: duration of invasive mechanical ventilation (days), severe bronchopulmonary dysplasia (BPD) [15], length of NICU stay (days), corrected age at discharge, need for home oxygen treatment, and mortality before 28 days after birth and before discharge.

### 2.2. Statistical Analysis

The included infants were categorized into the PPH and no-PPH groups. Baseline characteristics, respiratory morbidities, and in-hospital outcomes were compared between the two groups. Continuous variables were analyzed using the Wilcoxon rank-sum test, and categorical variables were analyzed using the chi-square test or Fisher’s exact test, as appropriate. Kaplan–Meier survival analysis was performed to compare survival between infants with and without PPH.

Multivariable logistic regression analysis was performed to assess the association of PPH and delayed PDA closure (≥21 days after birth) with the following neonatal outcomes: (a) adverse respiratory outcome (severe BPD and/or home oxygen treatment), (b) neonatal death, and (c) composite outcome (adverse respiratory outcome and/or neonatal death).

Cox proportional hazards regression analyses were performed to evaluate the associations between variables of interest and in-hospital mortality, using the length of hospital stay (days) as the time scale.

Statistical analyses were performed using SPSS ver. 28 (IBM Corp., Armonk, NY, USA), and *p*-values less than 0.05 were considered significant.

The study was approved by the institutional review board of our hospital (approval number: KC22WASI0911). The need for informed consent was waived due to the retrospective nature of the study.

## 3. Results

### Patient Characteristics

The baseline characteristics of the no-PPH and PPH groups are described in Table 1. BW was significantly lower in the PPH group than in the no-PPH group (median 931 g vs. 1070 g, *p* = 0.009), whereas GA did not significantly differ. The prevalence of SGA was higher in the PPH group than in the no-PPH group; however, the difference was not statistically significant. The prevalence of multiple births was greater in the no-PPH group than in the PPH group (40% vs. 27.4%, *p* = 0.035). PPROM duration was significantly longer (median 188 h vs. 18 h, *p* < 0.001) and onset was earlier (23.9 vs. 26.8 weeks’ gestation, *p* < 0.001) in the PPH group than in the no-PPH group. Sixty-eight of the 84 infants (81.0%) in the PPH group were affected by oligohydramnios.

Neonatal morbidities associated with respiratory status and pulmonary circulation are shown in Table 2. The PPH group had a significantly higher prevalence of air leak syndrome (19.0% vs. 8.1%, *p* = 0.004) and pulmonary hypertension (28.6% vs. 15.6%, *p* = 0.007). When analyzed specifically by the day of onset for pulmonary hypertension, early pulmonary hypertension occurred twice as often in the PPH group than in the no-PPH group in the whole study population (14.3% vs. 7.1%, *p* = 0.040). Meanwhile, the proportion of early and late pulmonary hypertension among those affected by pulmonary hypertension was similar regardless of the presence of PPH. Although the prevalence of hsPDA was similar between the PPH and no-PPH groups, the postnatal age at final treatment for PDA was significantly later in the PPH group than in the no-PPH group [median 33 vs. 19 days, *p* = 0.025]. The prevalence of delayed PDA closure was similar between the two groups when analyzed across the entire study population. When analyzed in those who were affected by hsPDA, the prevalence of delayed PDA closure was higher in the PPH group (11/16 = 68.8%) than in the no-PPH group (34/78 = 43.6%).

**Figure 2:**
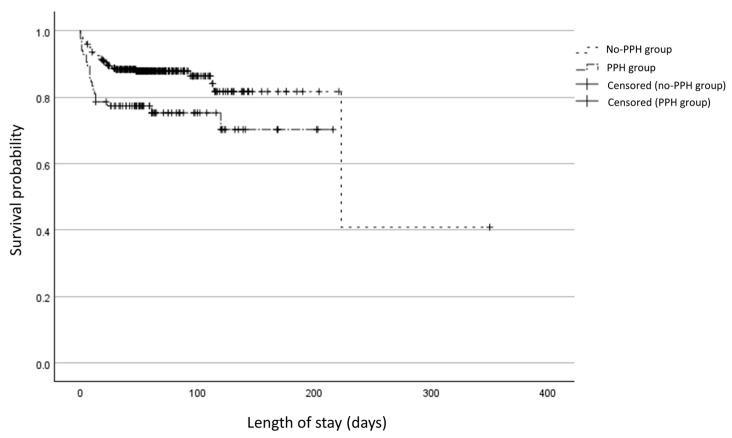
Kaplan–Meier survival analysis, PPH group vs. no-PPH group.

Table 3 shows the neonatal outcomes during the later days of NICU stay. While the duration of invasive mechanical ventilation, severe BPD prevalence, and need for home oxygen treatment did not significantly differ, mortality before NICU discharge was significantly higher in the PPH group than in the no-PPH group (25.0% vs. 13.2%, *p* = 0.009). Kaplan–Meier survival analysis (Figure 2) also revealed a significantly lower survival in the PPH group compared to the no-PPH group (log-rank χ^2^ = 7.046, *p* = 0.008). The median survival time was 160.6 days (95% CI, 139.8–181.5) in the PPH group, whereas it was 223.0 days (95% CI, 77.3–374.4) in the no-PPH group.

Multivariate logistic regression analysis was performed to evaluate the impact of early pulmonary hypertension and delayed PDA closure on neonatal outcomes (Table 4). BW (per 100 g), multiple births, and antenatal corticosteroid administration were added to adjust for confounding effects. Early pulmonary hypertension was associated with increased odds of neonatal death [odds ratio (OR): 11.165, 95% confidence interval (CI): 3.657–34.088] and composite outcomes (OR: 5.800, 95%CI: 1.263–26.633) when analyzed in the total cohort; however, when analyzed separately in the no-PPH and PPH groups, early pulmonary hypertension was significantly associated with neonatal death only (OR: 11.575, 95%CI: 2.988–44.833 and OR: 9.981, 95%CI: 1.334–74.647, respectively). Delayed PDA closure increased the odds of adverse respiratory and composite outcomes and decreased the odds of neonatal death in the total cohort. A separate analysis of the no-PPH group revealed similar results for adverse respiratory outcomes (OR: 4.929, 95%CI: 1.613–15.055), neonatal death (OR: 0.036, 95%CI: 0.003–0.383), and composite outcomes (OR: 3.320, 95%CI: 1.048–10.515). Delayed PDA closure was not associated with any outcome in the PPH group.

In the Cox proportional hazards regression analyses, six models were constructed (Table 5). Models 1–4 included baseline covariates (i.e., birthweight, multiple births, and air leak syndrome) that were also adjusted for in the multivariable logistic regression analyses, with additional inclusion of PPH, early pulmonary hypertension, or delayed PDA closure. Models 5 and 6 examined the interaction effects between PPH and early pulmonary hypertension, and between PPH and delayed PDA closure, respectively. Across all models, higher birthweight and absence of air leak syndrome were consistently and significantly associated with reduced mortality (*p* < 0.001). In contrast, none of the variables of interest—PPH, early pulmonary hypertension, or delayed PDA closure—showed statistically significant associations with mortality, either as independent predictors or in interaction terms.

## 4. Discussion

The present results suggest that neonatal death before discharge is more prevalent in infants with PPH than in those without. Early pulmonary hypertension was also associated with an increased risk of neonatal death, regardless of PPH status. Delayed PDA closure contributed to an increased risk of adverse respiratory outcomes and composite outcomes overall, but not specifically in infants affected by PPH. However, based on the Cox proportional hazards models, PPH, early pulmonary hypertension, and delayed PDA closure were not significant factors in terms of their time-to-event associations.

Amniotic fluid plays a pivotal role in the development of a normal lung. Mechanical compression of the fetal thorax due to loss of amniotic fluid hampers normal fetal breathing movements and generation of distended pressure by amniotic fluid [1,16], leading to interference with epithelial and endothelial development and reduction in lung cell size and the number of airway branches [17]. Animal studies have shown decreased collagen levels and elastic tissue development in hypoplastic lungs in cases of oligohydramnios [1,18]. The loss of amniotic fluid due to PPROM and oligohydramnios is linked to the pulmonary underdevelopment [19,20,21]. Despite a few reports suggesting diagnostic criteria [2,22,23], there are limited guidelines that concretely aid in diagnosing pulmonary hypoplasia in clinical settings. Therefore, prolonged PPROM latency and/or oligohydramnios were chosen to define PPH in our study.

Infants with PPH in our study exhibited a higher prevalence of several adverse outcomes, including neonatal mortality and respiratory morbidities like air leak syndrome, pulmonary hypertension, and BPD. The significantly higher prevalence of air leak syndrome observed in the PPH group may reflect greater pulmonary structural fragility, potentially linked to impaired alveolar development or relative surfactant deficiency due to reduced expression of the lamellar bodies of type II pneumocytes in the context of hypoplastic lung disease [24].

Both air leak syndrome and pulmonary hypertension in preterm infants are independently associated with an increased risk of neonatal death. In a recent study comparing neonatal outcomes of preterm VLBW infants of the Brazilian Network on Neonatal Research (Rede Brasileira de Pesquisas Neonatais, RBPN) vs. the Neonatal Research Network of Japan (NRNJ), the infants from RBPN exhibited 9.06-fold higher odds of mortality, with air leak syndrome being one of the leading associated factors [25]. In another recent report based on the National Kids’ Inpatient Database, the association of pneumothorax with increased odds for mortality was identified in all gestational age groups > 24 weeks (greatest in preterm infants 29–32 weeks) [26].

Impaired development of the pulmonary vasculature in association with pulmonary hypoplasia includes decreased size of the total pulmonary vascular bed, decreased number of vessels per unit of lung tissue, and increased pulmonary arterial smooth muscle, which are characteristic features leading to increased pulmonary vascular resistance [2]. This is the basis for investigating the effect of pulmonary vasodilators on suspected pulmonary hypoplasia in infants exposed to PPROM and/or oligohydramnios [3,27]. Although early pulmonary hypertension and neonatal death were both more prevalent in the PPH group than in the no-PPH group, early pulmonary hypertension itself was associated with increased odds of neonatal death, regardless of whether the infant had been affected by PPH. Arjaans et al. [7] reported that the persistent pulmonary hypertension phenotype, which corresponds to early pulmonary hypertension features in our study, was associated with a death rate of 42% before 36 weeks postmenstrual age (PMA).

Meanwhile, while early pulmonary hypertension showed consistent associations with neonatal death across both groups, the impact of delayed PDA closure was confined to the no-PPH group, and composite outcomes did not always follow the same directional trend. Our analysis indicated that PDA treatment occurred at a significantly later postnatal age in infants with PPH than in those without, indicating that delayed treatment of PDA in this setting may be due to increased pulmonary resistance and possibly early pulmonary hypertension in a subset of infants during the early days after birth. Since the clinical signs and symptoms of hsPDA are caused by left-to-right shunt flow, elevated pulmonary vascular resistance fundamentally hinders progression of hemodynamical compromise caused by “ductal steal.” Furthermore, in the case of pulmonary hypertension, which involves either complete or partial flow of the PDA shunt in the opposite direction (i.e., right-to-left shunt or bidirectional shunt, respectively), it may take more time to recover the left-to-right direction of the shunt and progress into a true hsPDA status. In other words, “delayed PDA closure” may be less delayed considering the relatively shorter duration of left-to-right shunt flow after restoration of the shunt direction. Recent studies have demonstrated that the duration of PDA shunt exposure contributes to adverse neonatal outcomes [10,28,29,30]. As such, it is plausible that PDA closure at ≥21 days of birth, which is shorter than the expected duration of actual hsPDA exposure, is not associated with adverse neonatal outcomes in infants affected by PPH.

On the other hand, delayed PDA closure in the no-PPH group was associated with increased odds of adverse respiratory outcomes and composite outcomes, and with decreased odds of neonatal death. This result does not represent a cause-and-effect relationship; it is logical to think that those who survived throughout the NICU stay were likely to experience delayed PDA closure. As the conservative approach to hsPDA became popular worldwide [31,32,33], a longer duration of PDA exposure was associated with the deterioration of neonatal respiratory conditions. For instance, Mirza et al. [29] and Deng et al. [28] both reported that prolonged exposure to hsPDA was associated with an increased risk of the composite outcome of BPD/death. The physiological explanation for their work is discussed in another recent publication [30], which demonstrated that in infants who underwent a later PDA closure, transcatheter hemodynamic variables indicated significantly higher pulmonary vascular resistance, highlighting the unfavorable impact of prolonged hsPDA exposure on the development of early pulmonary vascular disease. Our study results are consistent with previous studies in that the adverse effects of pulmonary vascular shear stress caused by prolonged ductal shunt flow may lead to an increased risk of bronchopulmonary dysplasia and/or pulmonary hypertension [29,34,35]. Taken together, these findings emphasize the importance of interpreting the impact of delayed PDA closure in the context of pulmonary vascular status. A physiologically tailored approach to PDA management with careful consideration of individualized pulmonary hemodynamics rather than uniform time-based criteria should be pursued.

It is noteworthy that in contrast to the logistic regression model and Kaplan–Meier survival analysis, Cox proportional hazards regression analyses did not demonstrate a statistically significant association between PPH, early pulmonary hypertension, or delayed PDA closure and mortality. This discrepancy can be partly explained by violations of the proportional hazards assumption, particularly for PPH and early pulmonary hypertension, whose mortality effects declined over time as indicated by significant time interaction terms (*p* < 0.001). Such time-varying effects may have led to the underestimation of their impact in the Cox models. From a clinical standpoint, this suggests that infants with PPH or early pulmonary hypertension face a heightened risk of early deterioration, underscoring the importance of early recognition and timely initiation of supportive therapies such as pulmonary vasodilators. Establishing clear screening thresholds for echocardiographic evaluation in infants with PPH may facilitate rapid diagnosis and early treatment, potentially mitigating the time-sensitive mortality risk.

By comparison, the absence of death events in the subgroup of infants with both PPH and delayed PDA closure likely precluded reliable estimation of hazard ratios in this group. Therefore, the findings from logistic regression and Kaplan–Meier analyses described above may offer more reliable guidance in this context.

Notably, both lower birthweight and the presence of air leak syndrome consistently showed significant time-dependent associations with mortality across all Cox models. While birthweight is a non-modifiable risk factor, this finding underscores the importance of early risk stratification and tailoring respiratory management accordingly. Optimizing ventilation strategies to minimize lung injury while ensuring adequate gas exchange and pulmonary and systemic circulatory balance is essential, particularly in extremely low birthweight infants. In addition, as air leak syndrome remains a major contributor to neonatal mortality as described above, clinicians should prioritize gentle ventilation approaches to reduce the likelihood of barotrauma. In addition, heightened vigilance is warranted to detect and respond promptly to sudden deterioration, especially in infants at high risk for pulmonary compromise.

Our study is meaningful in that we focused on two disease entities representing different pulmonary circulatory statuses (poor pulmonary flow vs. increased pulmonary flow) and investigated the consequential impact on specific respiratory outcomes in preterm infants with suspected pulmonary underdevelopment. It would be prudent to consider the baseline structural and volumetric characteristics of the lung and pulmonary vasculature when discussing “true hsPDA” exposure.

We acknowledge the limitations of this study, which include a retrospective design and a small sample size from a single institution. As in other retrospective studies, cause-and-effect relationships cannot be concluded from our study results, and only associative relationships can be inferred. In addition, the final treatment decision was made by the attending neonatologist, and the possibility of individual preferences may have introduced bias. However, the need for hsPDA treatment was fundamentally assessed in accordance with previously published criteria [36]; deviation from our unit strategy is likely to have been minimal. Finally, we used PPROM latency and the presence of oligohydramnios to define “presumed” and not “proven” pulmonary hypoplasia. Further, the degree of pulmonary underdevelopment in the infants who experienced prolonged PPROM latency and/or oligohydramnios may be variable, and classifying all these infants in one “PPH group” may obscure the potential impact of the severity of pulmonary underdevelopment. Future prospective studies using an appropriate diagnostic methodology to accurately define and stratify pulmonary hypoplasia in terms of severity should aim to complement the results of our study.

## 5. Conclusions

In conclusion, the impact of different pulmonary circulation statuses on neonatal mortality and respiratory morbidities in preterm infants varied depending on the presence of PPH. Delayed PDA closure may represent different durations of hemodynamically significant left-to-right shunt flow and thus differentially affect subsequent adverse respiratory outcomes in those with and without PPH. In comparison, early pulmonary hypertension appears to be the principal circulatory factor associated with neonatal mortality, regardless of PPH status. However, as such associations may vary over time, cautious interpretation and clinical application of these findings are warranted.

## Figures and Tables

**Table 1:** Baseline characteristics of the included patients according to PPH status.

	No PPH (n = 295)	PPH (n = 84)	*p*
Gestational age (weeks)	28.1 [26.1–30.1]	27.7 [25.4–30.1]	0.220
Birthweight (g)	1070 [820–1290]	931 [730–1227]	0.009
Male	144 (48.8%)	39 (46.4%)	0.700
SGA	48 (16.3%)	21 (25.0%)	0.067
Multiple births	118 (40.0%)	23 (27.4%)	0.035
Cesarean delivery	259 (87.8%)	76 (90.5%)	0.499
1 min Apgar	3 [2–5]	3 [1–4]	0.060
5 min Apgar	6 [5–7]	6 [4–7]	0.146
Maternal age (years)	34 [31–36]	33 [30–36]	0.156
Primiparity	179 (60.7%)	47 (56.0%)	0.436
Maternal diabetes	29 (9.8%)	5 (6.0%)	0.272
Maternal hypertension	46 (15.6%)	14 (16.7%)	0.812
Histologic chorioamnionitis	97 (33.3%)	37 (44.0%)	0.071
Conceived via ART	67 (22.7%)	16 (19.0%)	0.474
PPROM	94 (31.9%)	49 (58.3%)	<0.001
PPROM (hours) ^1^	18 [6–61]	188 [45–521]	<0.001
PPROM onset (weeks) ^1^	26.8 [25.3–29.0]	23.9 [22.6–27.6]	<0.001
PPROM before 25 weeks of gestation ^1^	20 (21.3%)	30 (61.2%)	<0.001
Oligohydramnios	0 (0.0%)	68 (81.0%)	<0.001
ACS administration	203 (69.0%)	67 (79.8%)	0.055

^1^ Analyzed in those with PPROM (94 in no PPH, 49 in PPH).

**Table 2:** Respiratory comorbidities and factors associated with pulmonary circulation status in the included infants.

	No PPH (n = 295)	PPH (n = 84)	*p*
RDS	265 (89.8%)	75 (89.3%)	0.885
Surfactant ≥ 2 doses	82 (27.9%)	20 (23.8%)	0.457
Air leak syndrome	24 (8.1%)	16 (19.0%)	0.004
Massive pulmonary hemorrhage	48 (16.3%)	18 (21.4%)	0.271
Pulmonary hypertension, total	46 (15.6%)	24 (28.6%)	0.007
Pulmonary hypertension depending on onset			
Early pulmonary hypertension *	21 (7.1%)	12 (14.3%)	0.040
Late pulmonary hypertension	25 (8.5%)	12 (14.3%)	0.113
HSPDA **	78 (26.9%)	16 (20.3%)	0.230
COX-I use	25 (32.1%)	2 (12.5%)	0.140 †
COX-I failure	8 (32.0%)	0 (0.0%)	>0.99 †
Surgical ligation	61 (78.2%)	14 (87.5%)	0.512 †
PNA at initial Tx.	18 [9–26]	33 [20–47]	0.09
CA at initial Tx.	29.7 [28.1–31.3]	30.9 [28.4–32.0]	0.199
PNA at final Tx.	19 [10–29]	33 [20–47]	0.025
CA at final Tx.	29.9 [28.1–31.4]	30.9 [28.4–32.0]	0.324
PDA closure ≥ 21 d	34 (11.5%)	11 (13.1%)	0.695

* Analyzed in 46 in no PPH, 24 in PPH. ** Analyzed in 290 in no PPH, 79 in PPH. † Fisher’s exact test.

**Table 3:** In-hospital outcomes of the included infants.

	No PPH (n = 295)	PPH (n = 84)	*p*
MV duration	11 [3–37]	10 [2–44]	0.890
Severe BPD *	95 (37.1%)	23 (35.9%)	0.862
Length of stay	56 [39–82]	53 [24–95]	0.382
CA at discharge	37.3 [35.7–39.9]	37.1 [34.9–40.0]	0.598
Home oxygen treatment **	89 (36.6%)	21 (36.8%)	0.976
Death before 28 days after birth	39 (13.3%)	13 (15.5%)	0.612
Death before discharge †	39 (13.2%)	21 (25.0%)	0.009

* Analyzed in 256 with no PPH and 64 with PPH. ** Analyzed in 293 in no PPH, 57 in PPH. (excluding infants transferred before 28 days of age). † Analyzed in 295 in no PPH, 84 in PPH. (excluding infants transferred to other hospitals).

**Table 4:** Multivariable logistic regression analysis for the effect of early pulmonary hypertension and delayed PDA closure (≥21 days of life) on neonatal outcomes.

	Early Pulmonary Hypertension		Delayed PDA Closure	
Included infants, total	*p*	OR (95%CI)	*p*	aOR * (95%CI)
Adverse respiratory outcome	0.293	2.129 (0.520–8.713)	0.002	4.849 (1.783–13.189)
Neonatal death	<0.001	11.165 (3.657–34.088)	<0.001	0.020 (0.002–0.176)
Composite outcome	0.024	5.800 (1.263–26.633)	0.044	2.906 (1.030–8.197)
No-PPH infants	*p*	OR (95%CI)	*p*	aOR * (95%CI)
Adverse respiratory outcome	0.310	2.379 (0.446–12.683)	0.005	4.929 (1.613–15.055)
Neonatal death	<0.001	11.575 (2.988–44.833)	0.006	0.036 (0.003–0.383)
Composite outcome	0.068	6.920 (0.867–55.267)	0.041	3.320 (1.048–10.515)
PPH infants	*p*	OR (95%CI)	*p*	aOR * (95%CI)
Adverse respiratory outcome	0.388	4.158 (0.164–105.578)	0.544	2.166 (0.179–26.279)
Neonatal death	0.025	9.981 (1.334–74.647)	>0.990	- **
Composite outcome	0.091	8.390 (0.710–99.135)	0.962	1.059 (0.099–11.360)

* Factors included in the model: birth weight (per 100 g), multiple births, antenatal corticosteroid administration, early pulmonary hypertension, and PDA closure ≥ 21 days after birth. ** In the PPH group, the mortality for delayed PDA closure was 0%.

**Table 5:** Cox proportional hazards regression analyses were used to evaluate the association between clinical variables and in-hospital mortality.

Model #	Variables	Hazard Risk (95%CI)	*p*-Value
Model 1	Birth weight (100 g)	0.703 (0.629–0.785)	<0.001
	Multiple births	0.669 (0.395–1.134)	0.128
	Air leak syndrome	0.263 (0.146–0.472)	<0.001
	PPH	0.912 (0.518–1.607)	0.768
Model 2	Birth weight (100 g)	0.704 (0.630–0.787)	<0.001
	Multiple births	0.671 (0.396–1.138)	0.130
	Air leak syndrome	0.261 (0.145–0.470)	<0.001
	PPH	0.915 (0.519–1.615)	0.778
	Delayed PDA closure	1.153 (0.910–1.462)	0.215
Model 3	Birth weight (100 g)	0.702 (0.627–0.785)	<0.001
	Multiple births	0.662 (0.391–1.121)	0.126
	Air leak syndrome	0.263 (0.146–0.474)	<0.001
	PPH	0.912 (0.518–1.608)	0.768
	Early pulmonary hypertension	0.983 (0.712–1.356)	0.915
Model 4	Birth weight (100 g)	0.705 (0.629–0.791)	<0.001
	Multiple births	0.673 (0.397–1.141)	0.132
	Air leak syndrome	0.259 (0.143–0.471)	<0.001
	PPH	0.916 (0.515–1.629)	0.775
	Delayed PDA closure	1.154 (0.910–1.465)	0.215
	Early pulmonary hypertension	0.986 (0.715–1.361)	0.936
Model 5	Birth weight (100 g)	0.707 (0.632–0.791)	<0.001
	Multiple births	0.663 (0.395–1.113)	0.128
	Air leak syndrome	0.263 (0.146–0.474)	<0.001
	PPH	0.911 (0.518–1.603)	0.765
	Early pulmonary hypertension	0.971 (0.704–1.339)	0.868
	PPH × Early pulmonary hypertension	0.973 (0.469–2.021)	0.943
Model 6	Birth weight (100 g)	0.713 (0.637–0.797)	<0.001
	Multiple births	0.661 (0.390–1.121)	0.123
	Air leak syndrome	0.266 (0.148–0.478)	<0.001
	PPH	0.920 (0.519–1.630)	0.781
	Delayed PDA closure	1.174 (0.924–1.492)	0.180
	PPH × Delayed PDA closure	0.805 (0.406–1.596)	0.531

## Data Availability

The data that support the findings of this study are available from the corresponding author, only upon reasonable request.

## Abbreviations

The following abbreviations are used in this manuscript:

ART	Assisted reproduction therapy
CA	Corrected age
COX-I	Cyclo-oxygenase inhibitor
hsPDA	Hemodynamically significant patent ductus arteriosus
MV	Mechanical ventilation
PNA	Postanal age
PPH	Presumed pulmonary hypoplasia
PPROM	Preterm premature rupture of membranes
SGA	Small-for-gestational age
Tx	Treatment
